# Evaluating information content of SNPs for sample-tagging in re-sequencing projects

**DOI:** 10.1038/srep10247

**Published:** 2015-05-15

**Authors:** Hao Hu, Xiang Liu, Wenfei Jin, H Hilger Ropers, Thomas F Wienker

**Affiliations:** 1 Department of human molecular genetics, Max-Planck Institute for Molecular Genetics, Berlin, 14195, Germany; 2 BlackBerry Deutschland GmbH, Bochum, 44799, Germany; 3 Systems Biology Center, National Heart, Lung, and Blood Institute, NIH, Bethesda, MD, 20892, USA

## Abstract

Sample-tagging is designed for identification of accidental sample mix-up, which is a major issue in re-sequencing studies. In this work, we develop a model to measure the information content of SNPs, so that we can optimize a panel of SNPs that approach the maximal information for discrimination. The analysis shows that as low as 60 optimized SNPs can differentiate the individuals in a population as large as the present world, and only 30 optimized SNPs are in practice sufficient in labeling up to 100 thousand individuals. In the simulated populations of 100 thousand individuals, the average Hamming distances, generated by the optimized set of 30 SNPs are larger than 18, and the duality frequency, is lower than 1 in 10 thousand. This strategy of sample discrimination is proved robust in large sample size and different datasets. The optimized sets of SNPs are designed for Whole Exome Sequencing, and a program is provided for SNP selection, allowing for customized SNP numbers and interested genes. The sample-tagging plan based on this framework will improve re-sequencing projects in terms of reliability and cost-effectiveness.

Next-generation-sequencing (NGS) has gained its ground in medical research in recent years, and NGS-based re-sequencing has become a prevalent procedure in revealing causative variants^1^,^2^. In the foreseeable future, re-sequencing-based diagnosis will be conducted on a routine basis, not only in research labs, but also in clinical facilities. This heralds an increasing number of samples, and unavoidably exacerbates one of the existing problems in re-sequencing projects, namely, sample mix-up, which will lead to wrong diagnosis. Although there has been hitherto no general investigation on its incidence, our in-house records and the anecdotal sources show the sample mix-up at a rate between 0.1% and 1%. This problem can originate in each step of a re-sequencing project, thus entailing step-wise quality control. The intuitive solution to sample mix-up is sample-tagging and sample-matching, pre and post re-sequencing procedure, respectively, which was simply explored and discussed in recent studies^3^,^4^,^5^. On the other hand, there are various studies showed that SNPs could provide information to distinguish different populations, cell lines and individuals^6^,^7^,^8^,^9^,^10^,^11^,^12^, and approaches for optimizing SNP selection have been proposed^7^,^8^,^11^,^13^,^14^,^15^. These approaches consider haplotypes and linkage disequilibrium, however, development of these approaches is usually earlier than the NGS era and the application in the NGS data has not been fully considered^7^,^8^,^11^,^13^,^14^. Also, most of these approaches are based on empirical procedures, thus lacking expansibility and robustness with the increasing haplotype data in diversified populations^7^,^8^,^11^,^15^. Therefore, a sophisticated SNP selection approach with a strict theoretical framework for sample-tagging in large-scale re-sequencing is still in need.

Any sample-tagging plan usually utilizes polymorphic markers, especially SNPs. The human genome is a hive of SNPs which accumulate in the long history of human evolution, diversifying individual genomes. There are more than 10 million SNPs in the human genome^16^, and the latest version of 1000 Genomes Project database (phase 3) even includes more than 78 million SNPs, but not all of them can serve as useful discriminative markers. On one hand, only SNPs at a certain level of minor allele frequency (MAF) can be used, e.g., SNPs with a very rare minor allele should not be engaged. On the other hand, varying SNP frequencies in different ethnic groups render it hard to define the versatile markers for all populations. Even not to mention that SNPs with alleles in linkage disequilibrium lead to the slowed ascent of cumulative discriminative capability. Because the cost of sample-tagging plan is approximately proportionate to SNP number, it is of significant value to illustrate the least number of SNPs required to distinguish different samples considering all the aforementioned scenarios.

Suppose the present world population is 7 billion and the two-state markers are used to label each individual, then there are at least 33 markers needed (2^33^ = 8.5 billion). In the same way, suppose the markers with three genotypes, e.g., homozygous wild-type, homozygous mutant, and heterozygous mutant, are used to label, the least number of markers is 21 (3^21^ = 10.4 billion). It is obvious that these plans can work only if the markers have the two characteristics: 1. each genotype of a marker should be equally frequent; 2. the genotypes of one marker should be statistically independent of all other markers. Since we know that these conditions do not hold in reality, there are definitely more markers needed. According to information theory, the information contained in the aforementioned 33 markers and 21 markers are identical, equal to 33 bits of Shannon-entropy^17^. In other words, any marker plan of labeling the world population has to provide the information content no less than 33 bits. Therefore, in this work, we construct a model to evaluate the information content of SNPs, so that the optimized SNP sets can differentiate individual genomes in a reliable and efficient way.

## Results

### Sample collection

Evaluation of SNPs’ information content depends on their MAF, so that it is favorable to obtain genotypes of individual genomes. Three large samples with genotypes are included in this work, i.e., 1,092 individuals of the 1000 Genomes Project^18^, 1,115 individuals of the International HapMap Project^16^, and 1,043 individuals of the Human Genome Diversity Project^19^. The allele frequencies of ~ 2 million variants in ~ 6,500 individuals from the Exome Sequencing Project are involved in part of the modeling^20^. The genotypes drawn from the in-house whole exome sequencing (WES) of 265 individuals are used in testing the model. In the ensuing sections of this paper, these notations are used, i.e., HapMap for the International HapMap Project, HGDP for the Human Genome Diversity Project, ESP for the Exome Sequencing Project, MPIMG265 for the in-house 265 individuals.

MPIMG265 is comprised of individuals from multiple ethnic groups, focusing on the populations from the Middle East, which are presently under-represented in the polymorphism databases. MPIMG265 includes 176 individuals from Iran, 9 from Pakistan, 6 from Turkey, 1 from Saudi Arab, and 73 from Germany and other European countries. For every individual, the informed written consent was obtained from each family. MPIMG265 samples are processed under the supervision of GENCODYS consortium, the European Commission Framework Program 7 (Health-241995). On the other hand, among these 265 individuals, there are 14 trios (42 individuals) and 4 quartets (16 individuals). The genotypes of these 265 individuals are extracted from the WES results, and the procedures of variant calling and genotyping are described previously^2^,^21^.

Only the coding SNPs on the autosomal chromosomes (RefSeq gene model, downloaded from UCSC Genome Browser in June of 2013) are considered in this work. There are 472,514, 1,093,534, 9,472 and 14,521 suitable SNPs collected from 1000 Genomes Project, ESP, HGDP, and HapMap, respectively.

In order to test the possible sample overlap between 1000 Genomes Project, HapMap, and HGDP, 2,236 coding SNPs shared by these three datasets are used to compare the individual genotypes mutually. Individuals with >95% identity in genotype are regarded as sample overlap. This test shows there is no sample overlap existing between 1000 Genomes Project, HapMap, and HGDP.

In order to test the model, the simulated genotypes of four major ethnic groups, i.e., African (AFR), European (EUR), East-Asian (ASN), and American (AMR), are generated according to the allele frequencies drawn from 1000 Genomes Project and HapMap. The simulation size for each ethnic group is 100 thousand individuals, and the simulated genotypes obey the Hardy-Weinberg equilibrium.

When evaluating the performance of the optimized SNP sets in differentiating individuals, random SNP sets with SNP numbers from 20 to 50 are each generated by 1,000 times. The random SNPs are all located on the autosomal chromosomes, with MAF between 0.4 and 0.5 according to 1000 Genomes Project and HapMap. These random SNP sets stand for an intuitive design which is expected to reach suboptimal performance, much better than random SNP sets without MAF restriction while inferior to the optimized SNP sets.

### Information content of single SNP in different datasets

**Figure 1:**
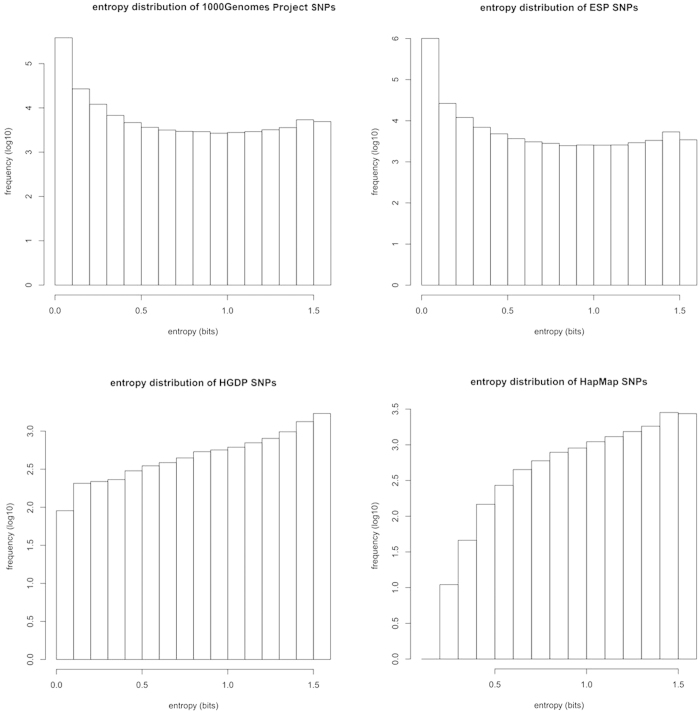
The entropy distribution of SNPs in 1000 Genomes Project, ESP, HGDP, and HapMap. The X-axis is the Shannon-entropy calculated for each SNP, the Y-axis is the frequency of entropy values in the specific database.

The information content, or Shannon-entropy of single SNP in 1000 Genomes Project, ESP, HGDP, and HapMap is calculated according to the variability of its three genotypes (homozygous wild-type, homozygous mutant, and heterozygous mutant). The information content is ranging from almost zero to as high as 1.585, which is the expected maximal entropy for a single SNP (Fig. 1). Since 1000 Genomes Project and ESP are both based on WES which covers all the coding SNPs indiscriminatingly, the majority of the SNPs have a low MAF, with entropies near zero accordingly. On the other hand, SNPs used in HGDP and HapMap are known to be common in population, therefore, the bulk of their entropies are larger than 1 bit. There are 6,745 SNPs shared by the four datasets, namely, 1000 Genomes Project, ESP, HGDP, and HapMap. In order to evaluate the consistency of SNP entropy from different datasets, the pairwise Pearson correlation coefficients are calculated (Table 1). The shared SNPs in the four datasets show high correlation in terms of entropy, which indicates the potential of choosing SNPs consistent in entropy in different datasets.

To refine SNPs by entropy consistency, SNPs are regarded as qualified if their entropies do not fluctuate in different datasets. Pairwise bootstrap tests are conducted to identify the qualified SNPs among 1000 Genomes Project, HGDP and HapMap, for the three datasets have different composition of ethnic groups. Only SNPs shared by 1000 Genomes Project, HGDP, and HapMap, which are equal in entropy in the 95% bootstrap percentile confidence interval, are remained. And SNPs whose mappability are not 1^21^ or GC-content beyond the expected interval of 0.35 - 0.55 are removed (the GC content scores are downloaded from UCSC Genome browser GC Percent (gc5Base) track). Consequently, there are 2,236 SNPs defined as the qualified SNPs for the subsequent procedures and their entropies are then re-calculated by the combined genotypes of 1000 Genomes Project, HGDP, and HapMap.

### Mutual information of qualified SNPs

**Figure 2:**
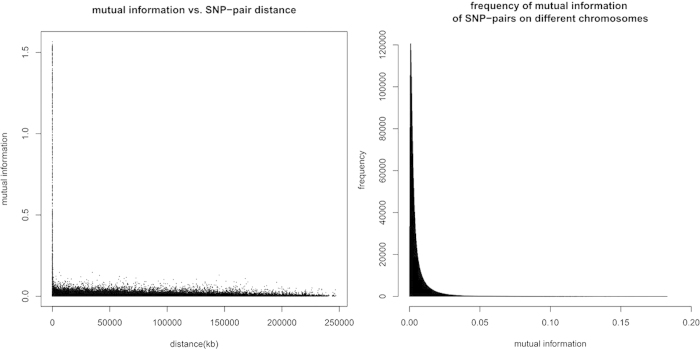
The mutual information distributions of SNP-pairs regarding distances and chromosomes.

In order to measure the genotype dependency between SNP pairs, the mutual information of each pair of the qualified SNPs is computed, based on the combined genotypes of 1000 Genomes Project, HGDP, and HapMap, altogether 3,250 individuals (Fig. 2). Large values of mutual information correspond to high mutual dependency, and thus a strong pattern of linkage disequilibrium (LD) between SNP pairs. Not very surprising, the SNP pairs close to each other have relatively higher mutual information. It is notable that the SNP-pairs on the same chromosomes have reduced mutual information with increasing distance but not dropping to zero. Also, the mutual information of SNP-pairs on different chromosomes, although much lower than those on the same chromosomes, are still not low enough to be totally neglected.

### Joint entropy of given-number SNPs

**Figure 3:**
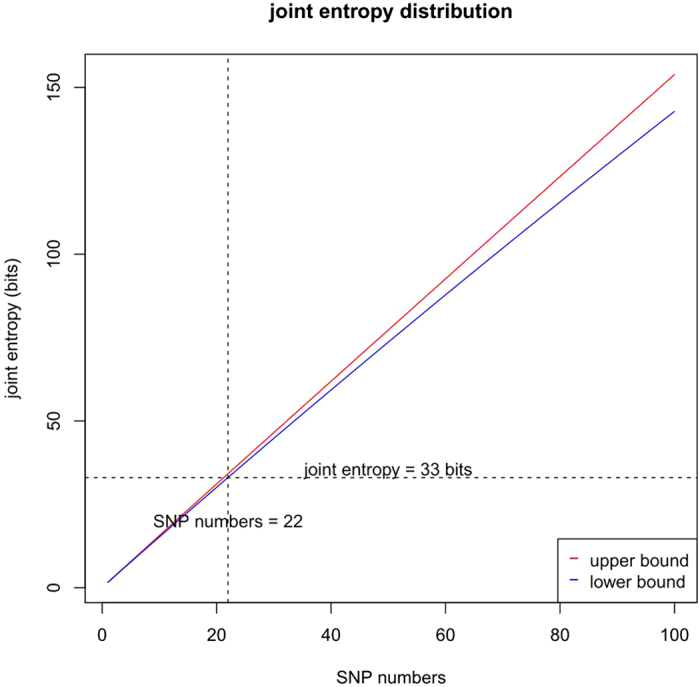
The lower bound and upper bound of joint entropy, i.e., *H*_L_ and *H*_U_, increase following the increasing SNP number from 1 to 100, with widening gap between *H*_L_ and *H*_U_. The joint entropy reaches 33 bits when the SNP number reaches 22.

Information theory tells us that the total information harbored in the multiple variables is measured by the individual entropies substracting the mutual information. Since the entropy of each SNP has been obtained, as well as the mutual information of SNP pairs, from the genotypes of 3,250 individuals, the total information, or joint entropy of all possible SNP combinations, can be calculated according to the aforementioned algorithm. It has been known that the exact value of joint entropy of more than 3 (*N* > 3) SNPs is difficult to calculate, but the upper and lower bounds can be estimated as *H*_*U*_ and *H*_*L*_. For the sets of SNPs with given number from 1 to 100 (*N*=1, ,100), the joint entropies are calculated for the maximal values, according to the aforementioned recursive algorithm. The gap between *H*_*U*_ and *H*_*L*_ grows as the number of SNPs increases (Fig. 3). Another interesting observation is that the joint entropy is 33 bits when the number of SNPs reaches 22. This indicates that at least 22 qualified SNPs are necessary for tagging the present world population.

### Expected Hamming distance and duality between individuals

There are two major concerns in designing discriminative set of SNPs: the expected Hamming distance, or mismatches, between a pair of individuals and the probability of duality, or total match.

A population of *L* individuals is considered. There are *S* genotypes for each SNP. A set of *N* independent SNPs is used for differentiating all *L* individuals in the population, assuming that all *S* genotypes are equiprobable for each SNP, thus reaching the maximal joint entropy. If the Hamming distance between any two individuals is greater than *t*, these two individuals are regarded as different. The *k*-combination of *n*-set is denoted as
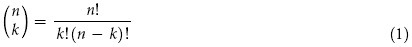
The *k*-permutation of *n*-set is denoted as
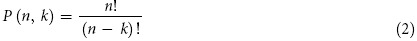


**Figure 4:**
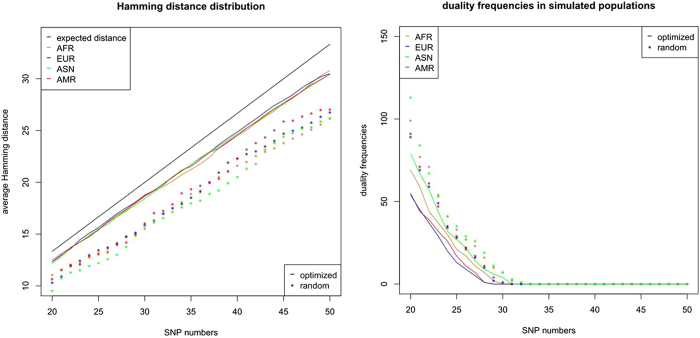
In the simulated populations (AFR, ASN, AMR, EUR), each of which has 100 thousand individuals, the Hamming distances and duality frequencies are generated by both the optimized sets of SNPs and the random sets of SNPs. The SNP numbers range from 20 to 50. The different populations are represented by different colors, and the lines stand for the optimized sets of SNPs and the asterisks stand for the average values of the random sets of SNPs.

The total number of individuals which can be differentiated by the *N*-SNP set is *S*^*N*^. The number of individual pairs which can be differentiated by the*N*-SNP set is *S*^*2N*^ . The number of individual pairs which has a Hamming distance of *k* in terms of the *N*-SNP set is
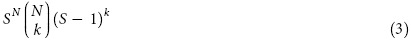
Hence the probability that two individuals having a Hamming distance of *k* in terms of the*N*-SNP set is
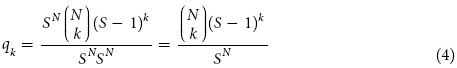
The probability that two individuals having a Hamming distance no more than *t* is
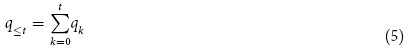
The expected Hamming distance between two individuals:
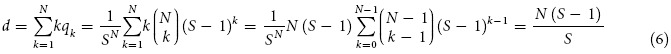
Thus, we have the expected Hamming distance 2*N*/3 in the case of*S* = 3 (Fig. 4).

The probability that any two individuals in a population of *L* individuals have a Hamming distance greater than *t* is approximated as
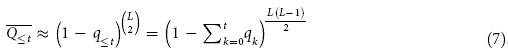
assuming 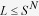.

The probability that at least two individuals in a population of *L* individuals have a Hamming distance no more than *t* is 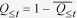, assuming *L* ≤ *S*^*N*^. This is difficult to calculate in the case of a large population size of *L*. We may use an approximation assuming *L* ≤ *S*^*N*^:
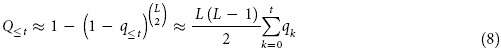
Thus, we have the probability 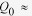
5.78e-10 when *L* = 7e9, *N* = 60, *S* = 3 and *t* = 0; and 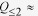
4.16e-6 when *L* = 7e9, *N* = 60, *S* = 3 and *t* = 2. Very interestingly, these results indicate that although the information content of an optimized set of 22 SNPs has the potential to label each individual in a population of 7 billion individuals, after assuming the independent distribution of genotypes, an optimized set of 60 SNPs can differentiate all 7 billion individuals with duality probability of 5.78e-10, even when tolerating 2 accidental SNP errors, the adjusted duality probability is still as low as 4.16e-6.

For example, if there is a population of 100 thousand individuals, and the optimized SNP sets are assumed independent in genotypes, according to our estimation of duality, the optimized set of 30 SNPs can give rise to a satisfactory duality probability of 2.4e-5.

### The optimized sets of SNPs show high Hamming distance and low duality in the simulated populations

According to the allele frequencies in HapMap and following the Hardy-Weinberg equilibrium, there are 100 thousand individuals generated as the simulated populations for four major ethnic groups, i.e., African (AFR), European (EUR), East-Asian (ASN), and American (AMR). In order to compare with the optimized SNP sets regarding the performance, for each of SNP numbers from 20 to 50, there are 1,000 random sets of SNPs generated. These random SNPs are chosen when their MAFs in 1000 Genomes Project and HapMap are between 0.4 and 0.5 and they are all located on the autosomal chromosomes. This reflects an intuitive plan which is supposed to reach suboptimal performance. According to the calculated Hamming distances in these simulated populations, the optimized SNP sets show superiority over the random SNP sets. For example, the average Hamming distance of an optimized set of given-number SNPs is larger than the random set by about 2. For example, an optimized set of 30 SNPs gives rise to the average Hamming distance of more than 18 in the simulation populations, while its random counterpart generates the average Hamming distance of around 16. And the duality frequencies of the optimized SNP sets are also much lower than the random ones, e.g., an optimized set of 30 SNPs can achieve very low duality frequency, which, even in the worst case of ASN, can be still less than 1 in 10 thousand (Fig. 4).

### The optimized sets of SNPs show high Hamming distance in the real samples

**Figure 5:**
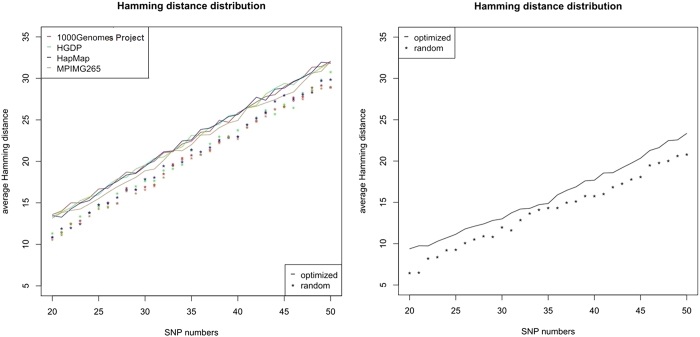
The Hamming distances are calculated based on the genotypes of 1000 Genomes Project, HGDP, HapMap, and MPIMG265 (left) and the relatives in MPIMG265 (right). The lines stand for the optimized SNP sets and the asterisks stand for the random SNP sets.

According to both the optimized set of SNPs and the random sets of SNPs, the Hamming distances are generated for 1000 Genomes Project, HGDP, HapMap, and MPIMG265 (Fig. 5). The differentiating capability of the optimized set of SNPs are obviously better than the random ones, and even in the relatives (42 pairs of parents-offspring or siblings), the optimized set of SNPs can still give rise to satisfactory discrimination. As mentioned in the previous sections, MPIMG265 is mainly composed of samples from a population under-represented in the present databases. The optimized set of SNPs performs still well in MPIMG265, although MPIMG265 is not involved in constructing the entropy model. On the other hand, it is well known that parent-offspring and siblings share half of their genetic information, so that the reduction of discriminative capability of SNP sets will be unavoidable. However, even in these harsh conditions, the optimized set of SNPs seems still applicable (Fig. 5).

### Software program for user-design SNP sets

A software program called SNP_Tagger has been developed for implementing the aforementioned algorithm. Users can download SNP_Tagger from https://sourceforge.net/projects/merap/files/SNP_Tagger20131220/. The software provides the 2,236 qualified SNPs as the starting menu, which have high and consistent information content in different datasets, and users can define the favorable SNP numbers and the gene list of interest. The default gene list includes all RefSeq genes on the autosomal chromosomes, assuming that most users will use WES. However, there are some users, especially clinicians, who may favor diagnostic panels including only part of the human gene repertoire, which thus can be defined as a curtailed gene list used by the program SNP_Tagger.

## Discussion

Re-sequencing is entering the clinical research as a routine diagnostic method, while the large amount of samples processed will unavoidably bring about the problem of sample mix-up. The manual re-identification at the end of each re-sequencing project by Sanger sequencing is laborious and not cost-effective. Therefore, it is worthwhile to label samples before entering the diagnostic pipeline. The intuitive and cost-effective solution is tagging samples with a set of SNPs, which are abundant in the human genome and can distinguish individual genomes. However, it is the skewed allele frequency which makes the bulk of SNPs unsuitable as tagging markers, then how to define a set of competent SNPs in terms of differentiating capability, and consistence in different sample sources, is of major concern. Our motivation to design the algorithm is to create the set of SNPs with minimal numbers while with highest differentiating capacity. The information content, or entropy of SNP set, measures the variability of the SNP combinations, thus correlated with its competence in labeling individuals. To the limit of our knowledge, this is the first attempt by using information theory to select a set of qualified SNPs for sample-tagging in re-sequencing studies. We confirm the optimized SNP sets outperform the random ones, and also offer software by which the users can generate the SNP sets of their interest, so as to develop the specific diagnostic panels which harbor only a fraction of the human genes. In the same principle, this SNP-tagging plan also has potential application in forensic science, in order to match the SNP fingerprints of the concerned subjects.

In practice, the more SNPs are employed in tagging samples, the higher is the differentiating capability. The cost of SNP-tagging should be less than the re-sequencing cost times the sample mix-up rate, otherwise it will also make sense to re-identify the mixed samples by re-sequencing *per se*. This is the reason why we postulate that only 60 SNPs can label a population of 7 billion individuals, and we recommend using 30 SNPs to label the thousands of individuals in a typical re-sequencing project. The tests in our study also show that by using the optimized set of 30 SNPs, the Hamming distances in pairs are large enough to distinguish members of the same family. We hope that the implementation of this algorithm will result in an enhanced reliability and validity of re-sequencing projects, relieving the practitioners from the consequences of inadvertent sample mix-ups.

## Methods

### Calculation of information content of a single SNP

The Shannon-entropy, or information content, of a single SNP, is calculated in this work according to the variability of its three genotypes, i.e., homozygous wild-type, homozygous mutant, and heterozygous mutant. The information content of a single SNP *X* is given by:
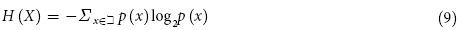
where *H*(*X*) is the information contained in a single SNP *X*, and 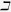 is the set of all three genotypes, i.e., 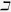 = {homozygous wild-type, homozygous mutant, heterozygous mutant}, and *p*(*x*) is the probability that the single SNP *X* has a specific genotype *x*, which belongs to 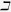.

Obviously, the maximal information content of a single SNP is 1.585 bits, which is achieved when the three genotypes are equiprobable. For a SNP with MAF = 0.5, when it has reached the Hardy-Weinberg equilibrium in a population, its information content is 1.5 bits.

In order to evaluate the consistency of information content in different sample sources, the bootstrap, namely, random sampling with replacement from the original dataset, is employed^22^. Given the genotypes of a single SNP from two sources with sample sizes of *n* and *m*, respectively, the bootstrap procedures are:

1. Draw a re-sample of size *n* with replacement from the first source and a re-sample of size *m* with replacement from the second source. Compute the entropy difference (*ΔH*) between the first re-sample and the second re-sample, regarding the single SNP.
2. Repeat this re-sampling process 1,000 times.
3. Construct a bootstrap distribution of the 1,000 *ΔH*.
4. The expected value of *ΔH* is 0, assuming the genotype of a single SNP is identical in the two sources. A single SNP is regarded as consistent if the observed *ΔH* is located in the 95% bootstrap percentile confidence interval.

Furthermore, in order to prevent the unsatisfactory performance of SNP detection in a variety of platforms, two factors have been considered for refining SNPs, i.e., mappability and GC-content. The mappability of a SNP measures the possibility of short-read alignment ambiguity due to homologous sequences, and mappability of 1 indicates no alignment ambiguity^21^. In our application, only the SNPs with mappability of 1 are remained for further consideration. GC-content determines the bias of detection, thus, only the SNPs in the regions of GC-content from 0.35 to 0.55 are retained in our study (the GC content scores are downloaded from UCSC Genome browser GC Percent (gc5Base) track).

### Calculation of mutual information of SNP pairs

Genotypes of any two SNPs in the human genome are in many cases not independent, because there might be linkage disequilibrium among their alleles, which thus reduces the cumulative discriminative capability of multiple SNPs. To measure the mutual dependency between SNP pairs regarding their genotypes, the mutual information (*I*) is used. The definition of mutual information between two SNPs *X* and *Y* is:
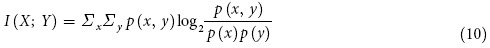
where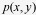 is the joint probability densities of *X* and *Y* regarding their three genotypes, and *p*(*x*) *p*(*x*) and *p*(*y*) are the marginal probability densities of *X* and *Y*, respectively, regarding their three genotypes. The higher the mutual information between *X* and *Y*, the more redundance in the information sum of *X* and *Y*.

### Estimation of combined information of multiple SNPs

The total information provided by *N* SNPs can be defined as the joint entropy 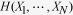, where 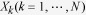 stands for the *k*-th SNP. Assuming that the joint entropy of the first 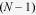 SNPs is known, the joint entropy of the *N* SNPs should be:
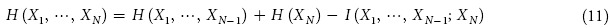
where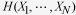 is the joint entropy of the *N* SNPs,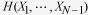 is the joint entropy of the first 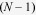 SNPs,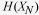 is the individual entropy of the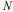-th SNP,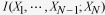 is the mutual information between the first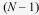 SNPs 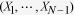 and the*N*-th SNP*X*_*N*_.

The exact value of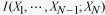 is difficult to calculate if *N* is large, but the upper bound and lower bound of the value can be estimated.

For the lower bound calculation, considering three SNPs, namely, *X*_1_,*X*_2,_*X*_3_, and using the chain rule for mutual information^17^, we have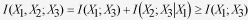, , since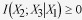. Similarly we have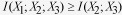. Extending to the multivariate mutual information, we have 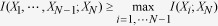.

For the upper bound calculation, considering three SNPs, namely, *X*_1_,*X*_2,_*X*_3_, and using the definition of mutual information, 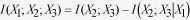, since the mutual information 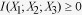, we have 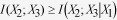. Substituting it into the chain rule for mutual information aforementioned, we have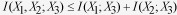. Extending to multivariate mutual information, we have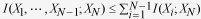.

Therefore, the lower bound (*H*_*L*_)and the upper bound (*H*_*U*_)of the joint entropy of *N* SNPs can be defined as:
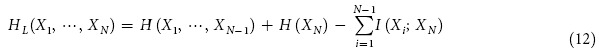

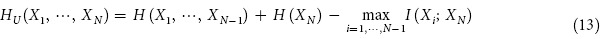


### Optimal set of a given number of SNPs in terms of combined information

In order to select an optimized set of given-number SNPs for sample-tagging, we have to maximize the combined information. The lower bound of the joint entropy 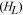 of a set of SNPs is used to estimate the combined information in this work. Given the individual entropy of each SNP and the mutual information of all SNP pairs, the question then is, how to find a set of SNPs, which has the maximal*H*_*L*_, or, has the highest discriminative capability. This problem is solved by an improved recursive algorithm. Suppose that there are *N* single SNPs as the candidates,

Step 1: when *N* = 1, take the 1,000 SNPs with the highest individual entropies;

Step 2: when*N* = 2, calculate the joint entropy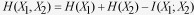. Take the leading 1,000 SNP pairs which have the largest joint entropies.

Step 3: when*N* = 3, calculate the joint entropy
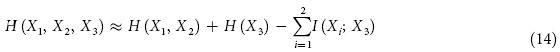
Hereby, (*X*_1_,*X*_2_) are defined in the 1,000 SNP pairs from the previous step. Take the leading 1,000 3-SNP sets which have the largest joint entropies.

Step *k*: when *N* = *k*, calculate the joint entropy
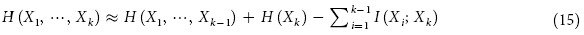
where 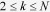. Hereby, 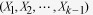
are defined in the 1,000 leading SNP sets from the previous step when *N* = *k*- 1. Take the leading 1,000*k*-SNP sets which have the largest joint entropies.

### Evaluation of SNP sets by Hamming distance

The optimized SNP sets should differentiate individuals most efficiently. This can be evaluated by the mismatches between sample pairs regarding the SNP sets. In information theory, the Hamming distance between two strings of equal length is the number of positions where the corresponding symbols are different^23^. Therefore, the Hamming distance is used to evaluate the performance of SNP sets in differentiation, i.e., the larger the Hamming distance, the better the performance. If the Hamming distance between two individuals is zero, the two individuals are claimed duality and the SNP sets could not differentiate between them, which indicates that the SNP set fails incidentally.

## Author Contributions

H.H., H.H.R. and T.F.W. initiated and coordinated this work. H.H. designed the scheme and collected the samples. L.X. and H.H. constructed and tested the mathematical models. J.W. provided the plan of assessing the model robustness in the different datasets. H.H., L.X. and T.F.W. wrote the manuscript.

## Additional Information

**How to cite this article**: Hu, H. et al. Evaluating information content of SNPs for sample tagging in re-sequencing projects. *Sci. Rep.* **5**, 10247; doi: 10.1038/srep10247 (2015).

## Figures and Tables

**Table 1:** The pairwise Pearson correlation coefficients among ESP, HGDP, HapMap, and 1000 Genomes Project, regarding the SNP entropy.

	**ESP**	**HGDP**	**HapMap**
1000 Genomes Project	0.893	0.965	0.957
ESP		0.84	0.874
HGDP			0.907
P-value <10^−16^			
